# Topology-Free EM Modeling and Analysis of Pixelated Microstrip Low-Pass Filters Under Comparable Footprint

**DOI:** 10.3390/mi17091060

**Published:** 2026-09-06

**Authors:** Jorge Davalos-Guzman, Jose L. Chavez-Hurtado, Lina M. Aguilar-Lobo

**Affiliations:** 1 División de Ingenierías, Subdirección de Investigación y Extensión, Centro de Enseñanza Técnica Industrial, Plantel Tonalá, Tonalá 45402, Jalisco, Mexico; jdavalos@ceti.mx; 2 Department of Electronics, Systems, and Informatics, Instituto Tecnológico y de Estudios Superiores de Occidente (ITESO), The Jesuit University of Guadalajara, Tlaquepaque 45604, Jalisco, Mexico; 3 Departamento de Computación e Industrial, Universidad Autónoma de Guadalajara, Av. Patria 1201, Zapopan 45129, Jalisco, Mexico; lina.aguilar@edu.uag.mx

**Keywords:** electromagnetic modeling, inverse design, microstrip filters, low-pass filters, pixelated structures, topology-free optimization, full-wave simulation

## Abstract

Pixelated electromagnetic representations enable topology-free numerical exploration of microwave layouts without enforcing a predefined circuit template. This work presents a simulation-based electromagnetic modeling and analysis framework for pixelated microstrip low-pass filters within a physical footprint comparable to that of a classical stepped-impedance reference. A binary metallization encoding is embedded in a fixed microstrip domain and explored through full-wave electromagnetic optimization under consistent material, excitation, and specification conditions. The objective is not extreme miniaturization or hardware prototyping, but to assess whether topology-free pixelated layouts can produce useful low-pass responses within the same general physical scale and to analyze the structural properties of the resulting feasible layouts. Across 20 independent optimization runs, 12 specification-compliant and geometrically unique metallization layouts were identified. Aggregate analysis of 360 single-pixel perturbations reveals topology-dependent sensitivity, while finite-width regularization of ideal corner contacts preserves modeled specification compliance after refined-mesh verification, with the suitable bridge width depending on the topology. The scope is deliberately limited to full-wave EM modeling and numerical analysis; experimental fabrication and measurement are left as a subsequent validation stage. These findings support topology-free pixelated EM modeling and analysis as a route for investigating non-obvious low-pass filter layouts under comparable footprint constraints.

## 1. Introduction

Microstrip filters remain essential components in RF and microwave systems because they provide frequency selectivity, interference suppression, and channel isolation in communication, radar, sensing, and front-end subsystems. Their design has traditionally relied on synthesis procedures derived from low-pass prototypes, impedance and admittance inverters, transmission-line transformations, and resonator coupling schemes, which are then translated into established physical layouts such as stepped-impedance sections, coupled-line structures, stub-loaded resonators, ring resonators, and related canonical topologies [1,2,3,4]. This classical framework offers physical interpretability, mature design procedures, and reliable routes from electrical specifications to manufacturable structures. At the same time, it implicitly constrains the designer to operate within a prescribed family of layouts whose topology is known in advance.

Under practical footprint limitations, this topological restriction becomes increasingly consequential. In planar microstrip implementations, improving selectivity, increasing filter order, extending stopband performance, or introducing transmission zeros often requires additional resonant sections, longer routing, folding, multilayer processing, or specialized compact-layout strategies [2,5,6,7,8,9,10]. A large body of work has therefore focused on compact microwave filters through stepped-impedance resonators, folded or meandered resonators, quasi-lumped implementations, and other layout-engineering techniques that preserve the logic of a known topology while reducing occupied area [5,6,7,8,9]. These methods are highly effective and remain central to microwave engineering practice. However, they mainly refine or compress a predetermined structure rather than asking whether the same physical footprint could host a functionally valid filter whose internal metallization is not tied to a conventional template.

Recent progress in simulation-driven and inverse electromagnetic design has broadened the way microwave structures can be synthesized. Instead of starting from a fixed circuit geometry and adjusting a relatively small set of continuous parameters, one may discretize the conductive region into many binary elements and let numerical optimization determine the final metallization pattern directly from the target response [11,12,13,14,15,16,17,18]. In this formulation, the design problem is no longer limited to perturbing a known topology; rather, it becomes a search over a much richer set of admissible layouts under fixed material, excitation, and boundary conditions. This shift is especially attractive in planar microwave design, where the available two-dimensional substrate area often defines the main physical resource and where classical layouts may not fully exploit the geometric freedom offered by that area.

Pixelated and free-form representations have already shown strong potential in several RF and microwave applications. They have been used in passive networks, vertical transitions, couplers, waveguide filters, antennas, and frequency-selective structures, often enabling compact, unconventional, or performance-enhanced realizations that would be difficult to derive from standard intuition-driven synthesis alone [19,20,21,22,23,24,25,26,27,28,29,30,31,32,33,34,35,36]. More directly related to filters, recent works have reported free-form or inverse-designed filter structures, including dual-band microstrip filters generated through generative models, tunable pixelated low-pass filters, ultracompact planar low-pass filters, and compact bandpass or notch-type pixelated filters synthesized by inverse design [10,19,37,38,39]. These results collectively indicate that pixelated EM design is not merely a numerical curiosity, but a viable synthesis paradigm for distributed microwave structures.

At the same time, most recent efforts have emphasized either aggressive miniaturization or faster inverse synthesis through machine learning and data-driven design. Those are important directions, but they leave room for a closely related question that is central to this work: what is gained by relaxing topology-specific assumptions when the physical footprint is kept comparable to that of a classical reference filter? In other words, if one does not pursue the smallest possible area, but instead retains a comparable envelope and removes the requirement that the layout follow a known stepped-impedance or resonator-based template, can the resulting pixelated structure still deliver a competitive low-pass response? If so, does the topology-free formulation reveal only one workable geometry, or does it admit more than one functional realization within the same domain? We do not frame this as a claim of universal superiority over classical synthesis, nor as a pure miniaturization exercise. Rather, we view it as a controlled investigation of whether topological freedom itself constitutes a meaningful design resource under a comparable footprint constraint.

This paper investigates topology-free pixelated microstrip filtering as a simulation-based electromagnetic modeling and analysis problem under a physical footprint comparable to that of a classical stepped-impedance reference. A sixth-order stepped-impedance low-pass filter is first used to define a physically meaningful benchmark in terms of substrate, excitation, frequency specifications, and overall geometric scale. A pixelated conductive domain is then embedded within a comparable envelope and explored through full-wave EM optimization using a 30-variable binary metallization encoding. The objective is not to achieve extreme area reduction or to establish a design-to-fabrication workflow, but to determine whether specification-compliant low-pass responses can emerge from layouts that are not forced to inherit a predefined internal topology. Accordingly, the scope is intentionally restricted to EM modeling, numerical optimization, and structural analysis of the resulting layouts rather than fabrication and measurement of a finalized hardware prototype.

The results show that topology-free pixelated realizations can indeed be obtained within the same general physical scale and can exhibit responses that are competitive with, and in some cases sharper than, the classical stepped-impedance reference. Independent optimization runs also reveal more than one specification-compliant metallization layout within the same design domain, indicating that the topology-free formulation does not collapse to a single canonical geometry, even though we avoid overinterpreting this observation until diversity is quantified more explicitly. To complement the response-level comparison, pairwise Hamming distance, pixel activation statistics, and aggregate single-pixel perturbation analysis are used to characterize geometric diversity, activation recurrence, and topology-dependent local sensitivity across the recovered feasible layouts. Finite-width regularization of ideal corner contacts is then examined under mesh refinement to assess whether representative layouts can be converted into finite-connectivity realizations without losing specification compliance.

Accordingly, the main contributions of this work are as follows. First, a topology-free pixelated EM modeling and analysis framework is formulated for microstrip low-pass filters under a footprint comparable to that of a classical stepped-impedance baseline. Second, full-wave optimization is used to show that removing topology-specific constraints can yield specification-compliant pixelated LPF layouts with competitive modeled response characteristics inside the same general physical envelope. Third, the work quantifies geometric diversity and topology-dependent single-pixel sensitivity across the recovered feasible set, and shows that ideal corner contacts in representative layouts can be regularized using finite-width conductive bridges while retaining modeled specification compliance after refined-mesh verification. Taken together, these results position topology-free pixelation here as a simulation-based framework for exploring and interpreting non-obvious microwave-filter layouts beyond prescribed topological templates, rather than as a complete hardware-development workflow.

## 2. Reference Filter, Pixelated EM Modeling Framework, and Evaluation Methodology

### 2.1. Classical Stepped-Impedance LPF Used as Reference

**Figure 1:**
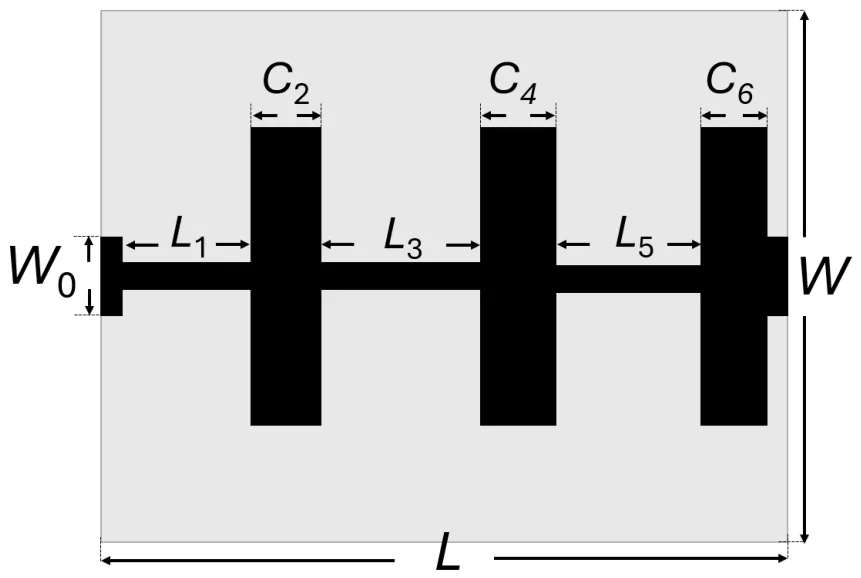
Geometry of the sixth-order stepped-impedance microstrip LPF adopted as the classical reference. $L_{1}$, $L_{3}$, and $L_{5}$ denote the high-impedance (inductive) sections; $C_{2}$, $C_{4}$, and $C_{6}$ denote the low-impedance (capacitive) sections. $W_{0}$ is the 50 Ω feed-line width, *W* is the substrate width, and *L* is the overall substrate length.

To assess the role of topology-free pixelated design under a controlled physical constraint, a classical stepped-impedance microstrip low-pass filter (LPF) is adopted as the reference structure. The purpose of this filter is not to serve as a strawman or as a fully optimized competing design, but rather to provide a physically meaningful benchmark in terms of substrate technology, excitation, electrical specifications, and overall geometric scale. The reference filter geometry is shown in Figure 1, and its final dimensions are summarized in Table 1.

The reference topology is a sixth-order stepped-impedance LPF composed of alternating high- and low-impedance microstrip sections. Initial dimensions were obtained from standard distributed low-pass filter design equations and subsequently adjusted through full-wave validation to account for distributed and fringing-field effects. The structure is implemented on an FR-4 substrate using the built-in material model in COMSOL, with relative permittivity $\varepsilon_{r}=4.5$, conductivity σ=0.004 S/m, and relative permeability $\mu_{r}=1$. The metallic traces are modeled as perfect electric conductors (PECs) to isolate geometry-driven electromagnetic effects and maintain consistent evaluation conditions across all designs considered in this work. The substrate thickness is 1.60 mm. Because the metallization is represented by PEC boundaries rather than a volumetric metal layer, no finite conductor thickness is assigned in the EM model.

The filter is excited through two identical lumped ports of characteristic impedance $Z_{0}=50\;\Omega$, located at the input and output faces of the substrate. The same port definition is retained throughout the paper for both the classical reference and the pixelated realizations. The final stepped-impedance layout consists of two 50Ω feed sections and six reactive sections with alternating impedance levels. After preliminary EM adjustment, the total reactive length is 26.104 mm, the total feed length is 1.60 mm, and the resulting overall trace length is 27.704 mm. The substrate width is defined as $W=7W_{0}$, with $W_{0}=3.048$ mm denoting the width of the 50Ω feed line; the same value is used as the pixel side length in Section 2.2, yielding W=21.336 mm.

Frequency-domain simulations were performed using the finite-element method. For visualization and the common comparative metrics reported later, the reference response was evaluated from 0.1 to 7 GHz using 51 uniformly spaced frequency points; its direct comparison with the representative pixelated responses is presented in Section 3.3. The electrical specifications adopted throughout this work are as follows: a passband from 0.1 to 3.5 GHz with $|S_{21}\left(f\right)|\;\geq 0.8$, a transition-band constraint from 3.8 to 4.2 GHz with $|S_{21}\left(f\right)|\;\leq 0.7$, and a stopband from 4.5 to 7 GHz with $|S_{21}\left(f\right)|\;\leq 0.2$. The optimization objective itself uses the band-specific discrete frequency grids detailed in Section 3.3. Under these conditions, the stepped-impedance filter satisfies the imposed spectral requirements and therefore serves as the classical reference used in the remainder of this study.

The use of this reference is important for two reasons. First, it provides a conventional microstrip LPF realization whose physical interpretation is clear and whose spectral response is well aligned with the target specifications. Second, it establishes a concrete benchmark for assessing whether a topology-free pixelated formulation can produce useful filtering responses within a comparable physical footprint, without inheriting the internal structure of a predefined filter layout.

### 2.2. Pixelated Design Domain Under Comparable Footprint

**Figure 2:**
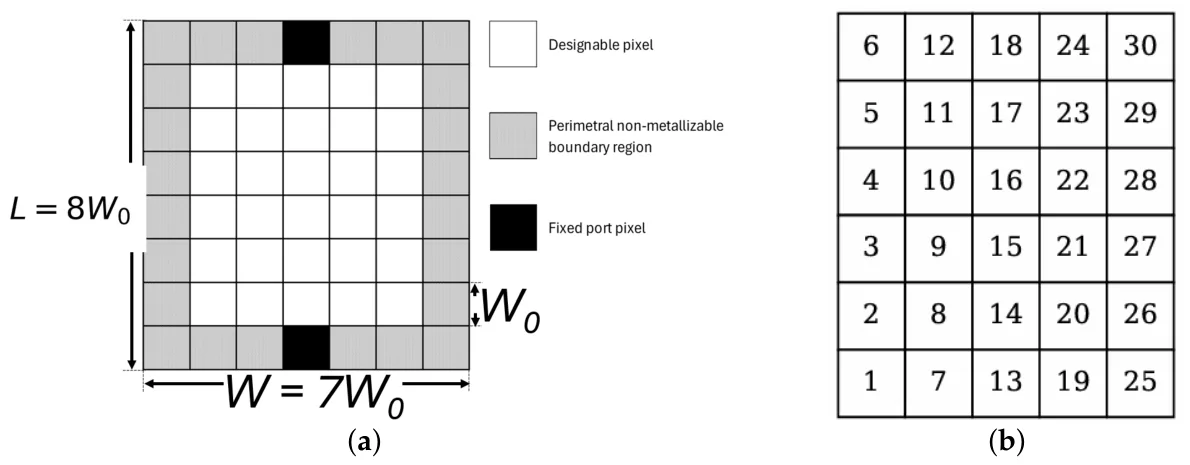
Pixelated design framework and binary encoding. (**a**) Discretized pixelated design domain embedded within the fixed microstrip envelope, showing the non-metallizable boundary region, the fixed port pixels, and the 6×5 active design region. (**b**) Pixel indexing is used to map the active metallization pattern to the optimization vector $x\in {\left\{0,1\right\}}^{30}$.

The topology-free formulation considered in this work is constructed by embedding a binary metallization domain within a fixed microstrip envelope of comparable physical scale to that of the classical reference. The discretized domain is shown in Figure 2a, and its geometric and encoding parameters are summarized in Table 2. Rather than prescribing a stepped-impedance topology, resonator arrangement, or coupling scheme a priori, the conductive pattern is allowed to emerge through the activation or deactivation of square metallization pixels within the available design region.

The full discretized domain is defined as an 8×7 grid of square cells of side length $W_{0}=3.048$ mm. This choice preserves the same substrate width used in the reference filter and yields a pixelated envelope of dimensions $8W_{0}\times 7W_{0}=24.384\times 21.336$ mm. The outer ring of cells is treated as a non-metallizable boundary region, while two fixed port pixels are permanently metallized to preserve the same input and output excitation configuration used in the reference structure. As a result, only the inner 6×5 subset constitutes the active design region, leading to a total of 30 binary design variables.

Each active pixel is assigned a binary state $x_{ij}\in \left\{0,1\right\}$, where $x_{ij}=1$ indicates the presence of metal and $x_{ij}=0$ denotes its absence. Adjacent active pixels share conductive edges whenever both are metallized, thereby forming arbitrary conductive paths and distributed metal regions inside the design domain. The corresponding binary encoding is illustrated in Figure 2b. Pixel indexing follows the deterministic column-wise ordering shown in Figure 2b, consistent with MATLAB R2025b (MathWorks, Natick, MA, USA) column-major ordering. This indexing defines the optimization vector.

(1) $$x=\mathrm{vec}\left(X\right)\in {\left\{0,1\right\}}^{30},$$

where $X\in {\left\{0,1\right\}}^{6\times 5}$ denotes the binary pixel matrix and vec(·) follows MATLAB R2025b column-major ordering. Under this formulation, the complete admissible design manifold is

(2) $$\Omega ={\left\{0,1\right\}}^{30},$$

which contains $2^{30}$ possible metallization patterns. No symmetry constraints, resonator templates, or circuit-topology rules are imposed inside the active region. In that sense, the formulation is topology-free: the design variables define metallization directly rather than geometric perturbations of a conventional microstrip filter architecture.

**Figure 3:**
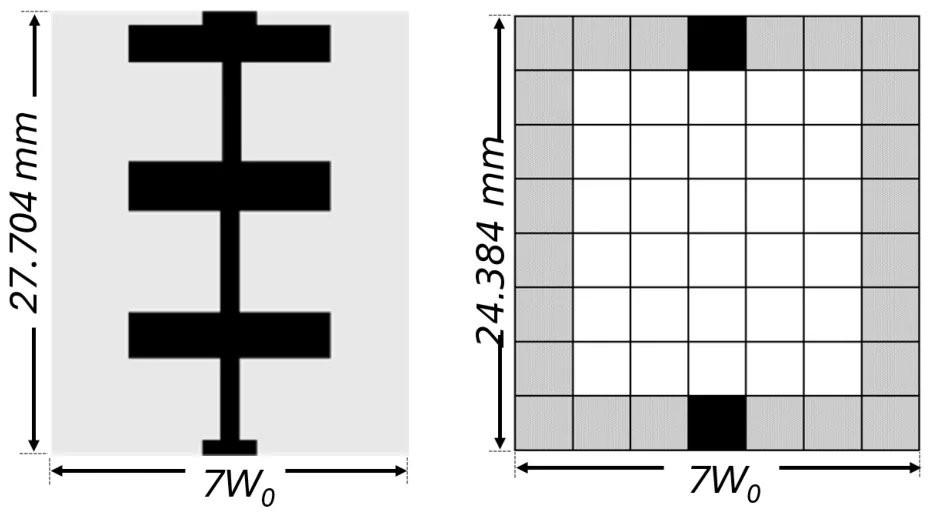
Geometric comparison between the classical stepped-impedance reference and the pixelated design domain under comparable footprint conditions. Both configurations share the same substrate width, while the pixelated formulation is confined to a comparable planar envelope used for topology-free EM exploration. In the pixelated panel, black cells denote the fixed metallized port pixels, white cells denote designable pixels, and gray cells denote the non-metallizable boundary region.

Although the pixelated domain is not identical to the stepped-impedance reference in internal organization, both operate under comparable footprint conditions. This comparison is illustrated in Figure 3. The two configurations share the same substrate width, and the pixelated envelope is confined to a similar longitudinal scale, with a total length of 24.384 mm compared with the 27.704 mm total trace length of the reference filter. The intention here is not to claim strict geometric equivalence, but rather to ensure that the pixelated formulation is explored under a physically comparable planar footprint.

This allows the subsequent comparison to focus on the effect of topological freedom, rather than on trivial differences arising from a radically different occupied area.

### 2.3. EM Model, Electrical Specifications, and Objective Function

Each candidate pixelated layout is evaluated through full-wave electromagnetic simulation using the same general EM environment adopted for the reference filter. All simulations are performed in COMSOL Multiphysics 6.3 (COMSOL AB, Stockholm, Sweden) in the frequency domain using the finite-element method. The substrate properties, port definitions, and boundary conditions are kept consistent throughout the entire design study. In particular, the pixelated structures use the same FR-4 substrate model, the same PEC metallization assumption, the same pair of 50Ω lumped ports, and the same surrounding-air and scattering-boundary treatment used for the classical stepped-impedance LPF. For the pixelated model, the surrounding-air domain is a rectangular block of dimensions 42.672×48.768×40 mm^3^, corresponding to 2W×2L×40 mm^3^. Its corner is located at (−10.668,−12.192,−20) mm relative to the substrate-base coordinate system, so that the block is centered laterally with respect to the substrate and spans from z=−20 mm to z=+20 mm. Scattering boundary conditions are applied at its outer boundaries. All optimization evaluations use the same mesh configuration stored in the COMSOL model; the effect of local mesh refinement around finite-width connections is assessed separately in Section 3.8.

This common EM setup defines a deterministic mapping from the binary metallization vector x to the corresponding transmission response:

(3) $$x⟼\left|S_{21}\left(f\right)\right|.$$

Thus, the comparison does not introduce changes in material model, port impedance, PEC assumption, or solver formulation; the principal design difference is the organization of the metallization within the respective planar geometries.

The same electrical specifications introduced in Section 2.1 are adopted for the pixelated designs. Performance is evaluated from the simulated transmission magnitude $\left|S_{21}\left(f_{k}\right)\right|$ sampled at discrete frequency points $f_{k}$. A band-based penalty formulation is used to quantify compliance with the passband, transition-band, and stopband requirements. In the implemented objective, the passband is sampled at 21 uniformly spaced points from 0.1 to 3.5 GHz, the transition band at 5 points from 3.8 to 4.2 GHz, and the stopband at 11 points from 4.5 to 7 GHz.

The passband requirement is

(4) $$\left|S_{21}\left(f\right)\right|\geq 0.8,\;f\in \left[0.1,3.5\;\mathrm{GHz}\right],$$

and the corresponding penalty term is defined as

(5) $$f_{\mathrm{pass}}\left(x\right)=\sum_{f_{k}\in \mathrm{PB}}\max\;\left(0,\;0.8-\left|S_{21}\left(f_{k}\right)\right|\right).$$

The stopband requirement is

(6) $$\left|S_{21}\left(f\right)\right|\leq 0.2,\;f\in \left[4.5,7\;\mathrm{GHz}\right],$$

with penalty

(7) $$f_{\mathrm{stop}}\left(x\right)=\sum_{f_{k}\in \mathrm{SB}}\max\;\left(0,\;\left|S_{21}\left(f_{k}\right)\right|-0.2\right).$$

The transition-band requirement is

(8) $$\left|S_{21}\left(f\right)\right|\leq 0.7,\;f\in \left[3.8,4.2\;\mathrm{GHz}\right],$$

with penalty

(9) $$f_{\mathrm{trans}}\left(x\right)=\sum_{f_{k}\in \mathrm{TB}}\max\;\left(0,\;\left|S_{21}\left(f_{k}\right)\right|-0.7\right).$$

The total objective function is then given by

(10) $$F\left(x\right)=f_{\mathrm{pass}}\left(x\right)+f_{\mathrm{stop}}\left(x\right)+f_{\mathrm{trans}}\left(x\right),$$

with equal weighting of the three terms. A pixelated layout is considered specification-compliant when

(11) F(x)=0,

meaning that all passband, transition-band, and stopband requirements are simultaneously satisfied.

This formulation is intentionally simple and fully response-based. It does not impose any explicit topological preference, connectivity constraint beyond what naturally arises from the binary metallization, or regularization favoring classical filter-like structures. Consequently, the search is driven only by electrical specifications under the fixed EM model and comparable footprint conditions described above.

### 2.4. Budget-Constrained Search Strategy

The binary formulation described above defines a combinatorial design manifold with $2^{30}$ admissible metallization patterns, while each candidate objective evaluation requires full-wave EM analyses over the three band-specific frequency grids defined in Section 2.3. Exhaustive exploration is therefore infeasible under realistic computational resources. To facilitate comparison with the classical stepped-impedance filter, Figure 3 shows the baseline geometry and the pixelated design envelope side by side. Both share the same substrate width $W=7W_{0}$, while the pixelated domain occupies a slightly shorter longitudinal footprint $\left(8W_{0}=24.384\;\mathrm{mm}\right)$ than the baseline total trace length $\left(27.704\;\mathrm{mm}\right)$. The domains are therefore comparable in size, so performance differences cannot be attributed to trivial geometric scaling.

The nominal design manifold contains $2^{30}$ = 1,073,741,824 binary metallization patterns. Accordingly, a budget of 300 candidate EM evaluations samples at most (300/$2^{30}$) × 100 ≈ 2.79 × $10^{-5}$% of the nominal space in each run. This comparison quantifies the scale of the search and does not imply uniform sampling of the manifold. In particular, the present study does not assume that 300 evaluations adequately sample the $2^{30}$ design space. Rather, 300 evaluations define a deliberately limited and identical full-wave computational budget under which the repeated discovery of specification-compliant layouts is assessed.

To explore this domain, a population-based evolutionary search strategy is adopted, and the optimization problem is formulated as

(12) $$\min_{x\in {\left\{0,1\right\}}^{30}}F\left(x\right),$$

where F(x) is the specification-violation metric defined in Equation (10).

The search is implemented using MATLAB’s genetic algorithm solver, with the configuration summarized in Table 3. The GA is selected because the design variables are naturally binary and the implemented full-wave objective is treated as derivative-free; no claim of optimizer superiority is made here. A bitstring representation is used for the binary design vector, the population size is set to 15, and two mutation probabilities, $p_{m}=0.05$ and $p_{m}=0.10$, are considered in independent runs. These values are used as two exploratory mutation regimes rather than as tuned or globally optimal hyperparameters. Under the 30-bit encoding, the corresponding expected mutation loads are $30p_{m}=1.5$ and 3.0 bit changes per offspring, respectively, providing a controlled comparison between a milder and a stronger binary perturbation level while keeping all other GA settings unchanged. The computational budget is fixed at $N_{EM}=300$ candidate EM evaluations per run as a common cost cap, where one candidate evaluation denotes one objective-function call over the three band-specific full-wave sweeps. The cap is not a convergence criterion. A run terminates when the specification-compliance condition in Equation (11) is met, when the stated GA stopping conditions are triggered, or when the evaluation cap is reached. Ten independent runs are performed for each mutation setting to obtain a representative set of feasible pixelated realizations for subsequent analysis.

For reproducibility, Algorithm 1 formalizes the implemented optimization sequence. It makes explicit the binary initialization, the three-band full-wave evaluation of each candidate, stochastic-uniform selection, elite preservation, scattered crossover, uniform bit-flip mutation, and the stopping conditions. The GA therefore acts only as the search engine over the binary metallization vector; every fitness value is obtained directly from the full-wave EM model through Equation (10).

**Figure 4:**
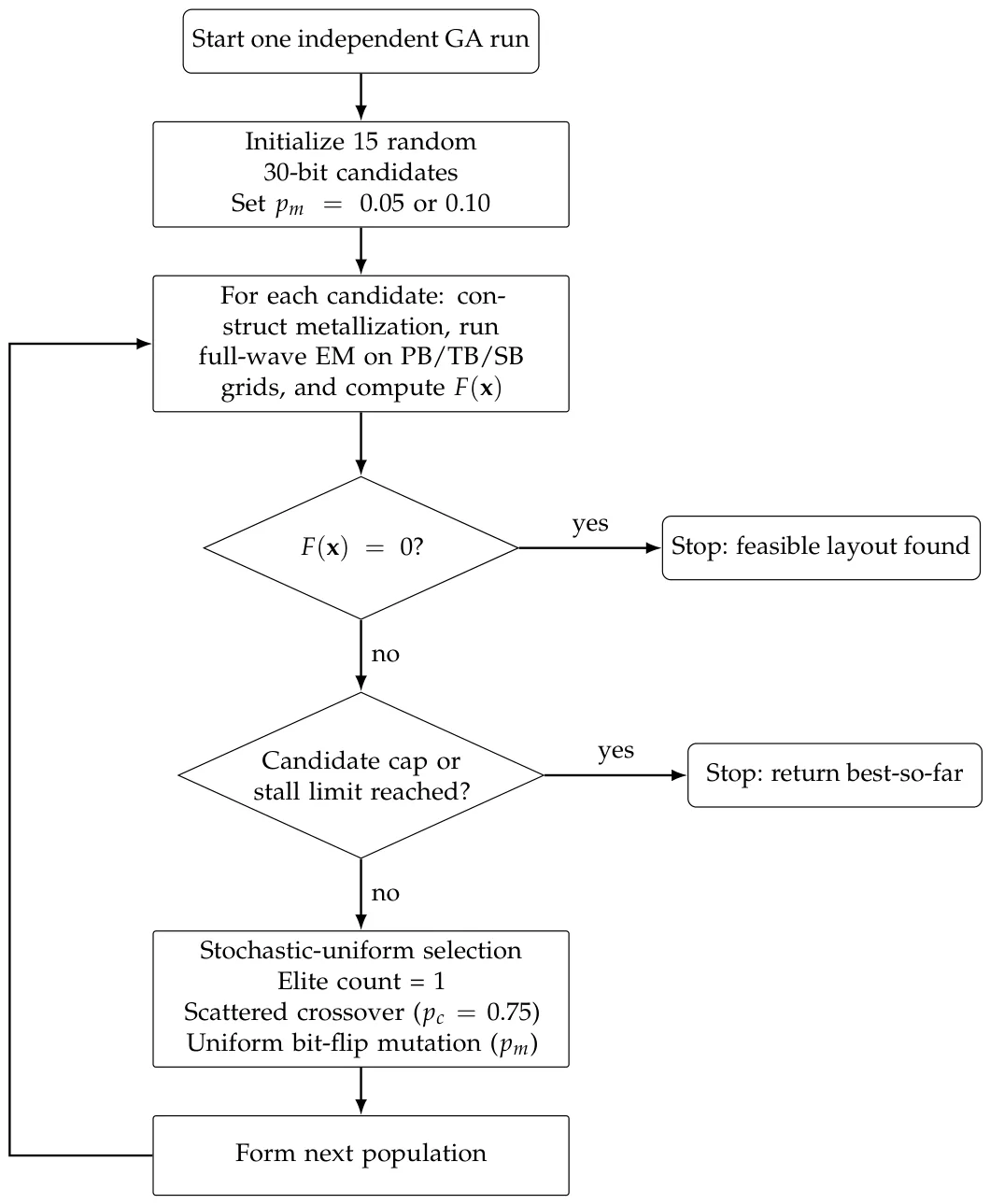
Optimization workflow used in each independent run. The GA operates on the 30-bit metallization vector, while every candidate fitness value is obtained directly from the three-band full-wave EM evaluation. The run stops upon specification compliance, exhaustion of the 300-candidate EM cap, or activation of the numerical stall criterion.

Figure 4 presents the same procedure graphically and highlights the separation between the binary GA operations and the direct full-wave candidate evaluation. No surrogate model or topology-specific synthesis stage is inserted in the optimization loop. Compliance is checked from F(x), while the candidate-evaluation cap and numerical stall criterion provide independent termination safeguards.

Together, Table 3, Algorithm 1, and Figure 4 separate the solver configuration from the candidate-evaluation sequence and provide a reproducible description of the implemented search without implying that the selected GA settings are globally optimal.
**Algorithm 1** Budget-constrained topology-free full-wave GA search**Require:** Binary dimension N=30; population size $N_{p}=15$; elite count $N_{e}=1$; crossover fraction $p_{c}=0.75$; mutation probability $p_{m}\in \left\{0.05,0.10\right\}$; candidate EM cap $N_{\mathrm{EM},\max}=300$**Ensure:** Best binary metallization vector found in the run1:Initialize a random bitstring population $P=\{x^{\left(1\right)},\ldots ,x^{\left(N_{p}\right)}\}$2:Set candidate-evaluation counter $N_{\mathrm{EM}}\leftarrow 0$3:**while** no stopping condition is active **do**4:    **for all** x∈P requiring fitness evaluation **do**5:        Apply x to the 30-pixel metallization domain6:        Run the full-wave EM model on the passband grid (21 points, 0.1–3.5 GHz)7:        Run the full-wave EM model on the transition-band grid (5 points, 3.8–4.2 GHz)8:        Run the full-wave EM model on the stopband grid (11 points, 4.5–7 GHz)9:        Compute F(x) from Equations (5), (9), and (7)10:        $N_{\mathrm{EM}}\leftarrow N_{\mathrm{EM}}+1$11:        **if** F(x)=0 **then**12:           **return** x             ▹ specification-compliant solution13:        **end if**14:        **if** $N_{\mathrm{EM}}\geq N_{\mathrm{EM},\max}$ **then**15:           **return** best-so-far x          ▹ candidate EM cap reached16:        **end if**17:    **end for**18:    **if** stall criterion is met **then**19:        **return** best-so-far x       ▹ 15 stall generations; tolerance $10^{-5}$20:    **end if**21:    Select parents by stochastic-uniform selection22:    Preserve the best $N_{e}=1$ individual unchanged23:    Generate offspring using scattered crossover with fraction $p_{c}=0.75$24:    Apply uniform bit-flip mutation with probability $p_{m}$25:    Form the next population P26:**end while**

## 3. Results—Pixelated LPF Realizations Under Comparable Footprint

### 3.1. Search Outcomes and Objective Evolution Under the Two Mutation Settings

To connect the representative layouts analyzed below with the stochastic search process, the outcomes and best-so-far objective histories of all 20 independent runs were examined. Ten runs were performed for each mutation probability, $p_{m}=0.05$ and $p_{m}=0.10$, under the same cap of 300 candidate EM evaluations. Six runs in each group satisfied Equation (11), producing 12 specification-compliant and geometrically unique layouts in total. The remaining eight runs reached the evaluation cap without satisfying all three spectral masks. Table 4 summarizes the empirical success rates, 95% Wilson confidence intervals, and the candidate-evaluation count at which F=0 was first reached in the successful runs.

**Figure 5:**
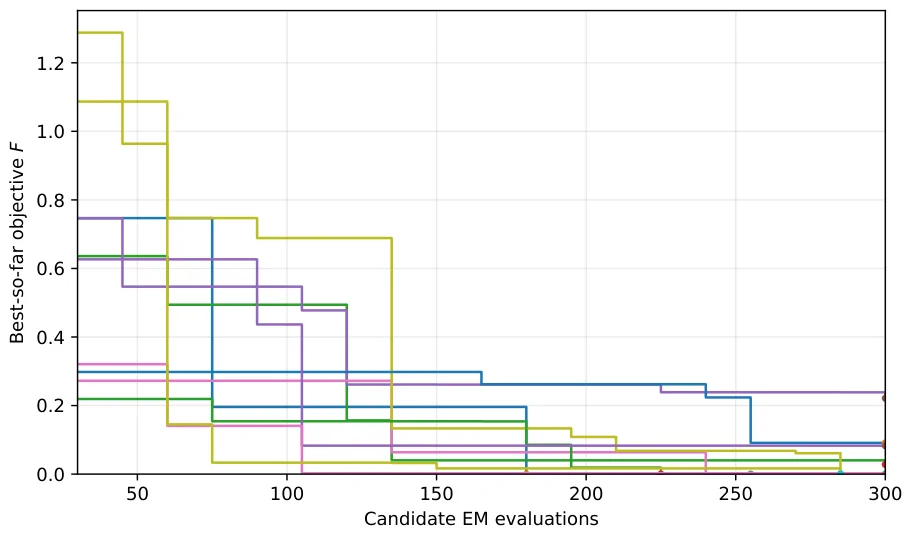
Best-so-far objective evolution for the ten independent runs with $p_{m}=0.05$. Each trajectory corresponds to one random seed and is plotted against the actual number of candidate EM evaluations. Different colors are used only to distinguish individual random-seed trajectories and carry no additional physical meaning. Six trajectories reach F=0 within the 300-evaluation budget; the four remaining runs terminate at the budget with positive residual objective values.

**Figure 6:**
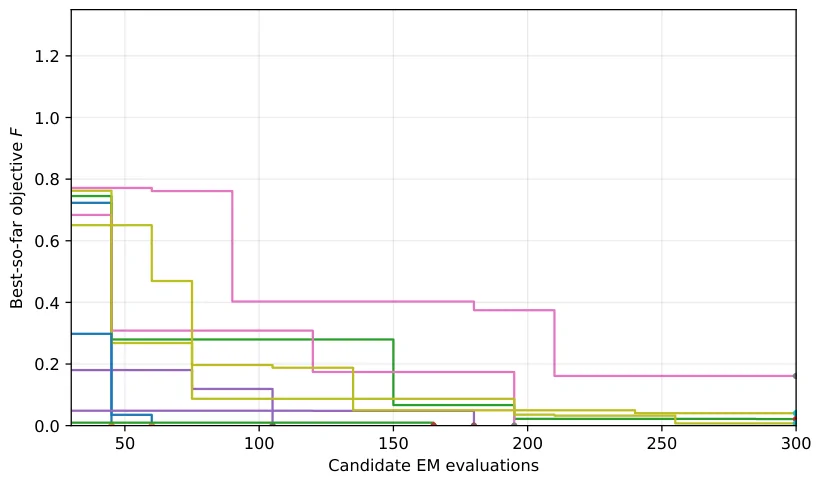
Best-so-far objective evolution for the ten independent runs with $p_{m}=0.10$. Different colors are used only to distinguish individual random-seed trajectories and carry no additional physical meaning. Six trajectories reach F=0 within the 300-evaluation budget, with feasible layouts appearing earlier than in the $p_{m}=0.05$ group for the present set of seeds. The four unsuccessful runs retain positive residual objective values at the budget limit.

Figure 5 and Figure 6 show the logged best-so-far value of the response-violation objective as a function of the actual candidate EM evaluation count for every independent run. Each trace ends at the evaluation at which the run stopped; therefore, successful traces terminate when F=0 is first reached, whereas unsuccessful traces extend to the 300-evaluation cap. These histories provide a direct view of the search progress without interpreting the budget as a formal convergence threshold.

The convergence histories also clarify what can and cannot be inferred from the selected budget. For $p_{m}=0.05$, the four unsuccessful runs ended with best objective values between 0.0281 and 0.2216; for $p_{m}=0.10$, the corresponding range was 0.00734–0.1613. Thus, some runs continued to approach the feasible boundary without reaching F=0 before the cost cap. Increasing the budget could therefore change the observed success rate, and no claim is made that 300 evaluations are sufficient for convergence or representative sampling of the complete combinatorial space. The budget is used instead as a controlled resource constraint that makes the 20 stochastic searches directly comparable.

**Figure 7:**
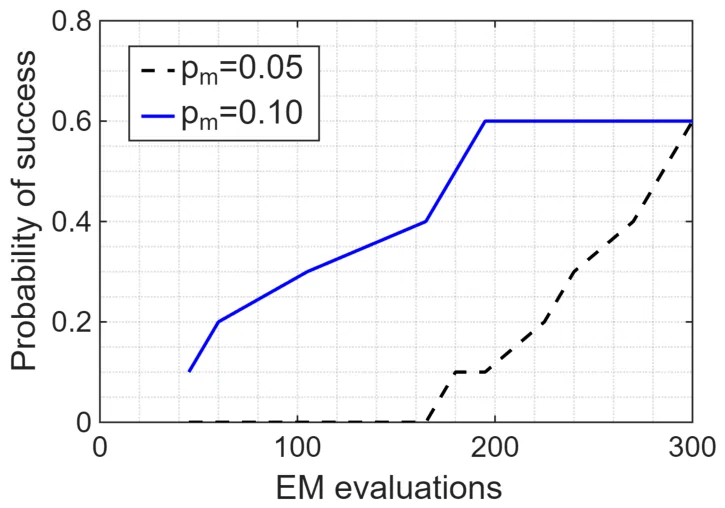
Empirical success probability as a function of the candidate EM evaluation budget for two mutation probabilities. Each curve represents the cumulative fraction of independent runs that reached a specification-compliant filter design by the indicated number of candidate evaluations. Both mutation settings achieved 6 successful runs out of 10 by 300 evaluations; however, the $p_{m}=0.10$ regime produced feasible layouts earlier and maintained a higher success fraction over most of the available evaluation budget.

The empirical cumulative success curves in Figure 7 provide a complementary budget-oriented view. For $p_{m}=0.10$, feasible layouts appeared substantially earlier and the terminal 0.60 success level was reached before 200 candidate EM evaluations. For $p_{m}=0.05$, the same final success level was reached only at the 300-evaluation limit. Because each group contains only ten independent runs, the identical 60% point estimates carry broad and substantially overlapping 95% confidence intervals; the timing difference is therefore interpreted descriptively rather than as evidence of statistically established superiority of one mutation probability.

Accordingly, the observed success rates characterize only the behavior of the present GA configuration under the stated 300-evaluation budget and should not be interpreted as an estimate of the density of feasible layouts within the full $2^{30}$ design space. Likewise, the earlier discovery observed for $p_{m}=0.10$ does not establish universal superiority of that mutation setting; it indicates faster discovery only for the present binary domain, initialization procedure, stopping rule, and evaluation budget.

#### Sensitivity to the Mutation Probability

The existing 20-run experiment also provides a controlled two-level sensitivity check for the mutation probability because the two groups differ only in $p_{m}$, while the population size, selection and crossover operators, stopping rules, objective function, and 300-evaluation cap are identical. The two tested values correspond to expected mutation loads of 1.5 and 3.0 bits per offspring for the 30-bit representation. Both settings produced the same terminal success rate, 6/10, so a two-sided Fisher exact test gives p=1.00 for the difference in success proportions.

The successful runs nevertheless reached feasibility descriptively earlier for $p_{m}=0.10$ (median 135 evaluations) than for $p_{m}=0.05$ (median 270 evaluations). To avoid conditioning the comparison only on successful runs, time to first F=0 was also analyzed, with the four unsuccessful runs in each group treated as right-censored at the 300-evaluation limit. A two-sided log-rank comparison gives p=0.45, which does not provide evidence of a statistically established difference in time to feasibility for this small sample. Accordingly, the results support only a local sensitivity interpretation: within the two tested mutation regimes, the success probability is indistinguishable and the apparent timing advantage of $p_{m}=0.10$ should be regarded as descriptive.

A formal optimization of GA hyperparameters would require a broader set of mutation probabilities and, preferably, a dedicated tuning protocol with additional independent full-wave runs. Such a campaign would shift the focus from topology-free EM synthesis to optimizer calibration, and is therefore beyond the scope of the present study. The values $p_{m}=0.05$ and 0.10 are consequently reported as exploratory settings rather than optimal choices; systematic hyperparameter optimization is identified as a separate direction for future work.

### 3.2. Representative Specification-Compliant Pixelated Designs

The topology-free formulation yielded multiple specification-compliant low-pass filter realizations within the fixed pixelated domain. Three designs are selected as representative examples because their original optimization vectors can be reproduced directly from the 30-bit encoding, and their full-wave responses reproduce the stored response curves used for the manuscript comparison. These designs correspond to the successful runs denoted here as Pixelated LPF 1, Pixelated LPF 2, and Pixelated LPF 3.

**Figure 8:**
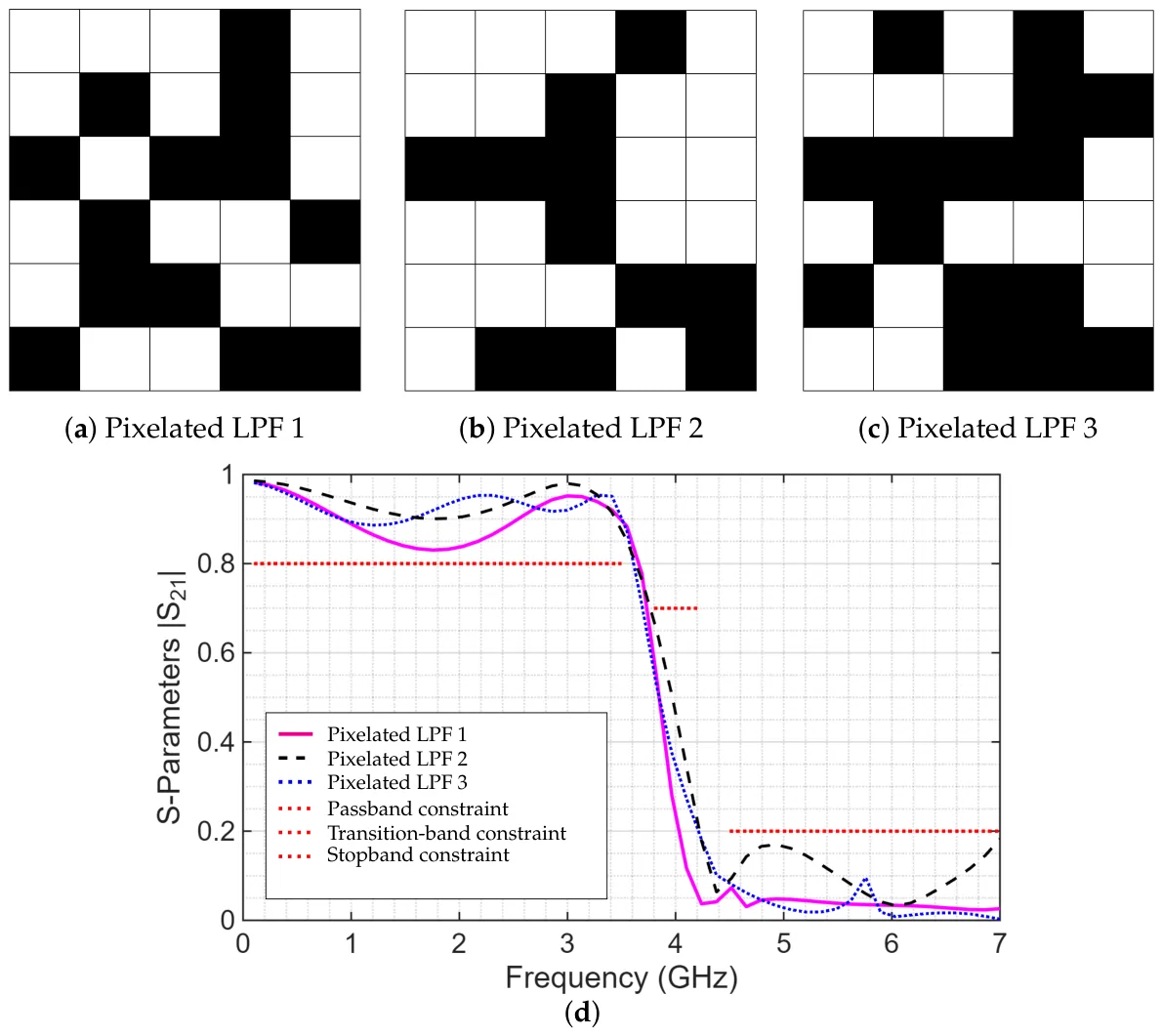
Representative topology-free pixelated microstrip low-pass filter realizations under comparable footprint conditions. (**a**–**c**) Verified binary metallization patterns corresponding to the three response curves, displayed with the same physical orientation as Figure 2b; black cells denote metallized pixels and white cells denote non-metallized pixels. (**d**) Simulated $|S_{21}|$ responses of Pixelated LPF 1 (magenta solid), Pixelated LPF 2 (black dashed), and Pixelated LPF 3 (blue dotted). Red dotted segments denote, over their respective frequency intervals, the passband, transition-band, and stopband specification masks. The response curves shown in (**d**) use a uniform 51-point display sweep from 0.1 to 7 GHz; strict compliance is evaluated on the original objective-function frequency grids.

For clarity, the three binary metallization maps are redrawn directly from their verified design vectors in Figure 8. The accompanying response panel is evaluated over a uniform 51-point sweep from 0.1 to 7 GHz for visualization and comparative metrics. Specification compliance itself is determined using the discrete frequency sets of the optimization objective: 21 points in 0.1–3.5 GHz, 5 points in 3.8–4.2 GHz, and 11 points in 4.5–7 GHz. All three representative designs satisfy Equation (11) on those original objective grids.

The three layouts are not perturbations of a common stepped-impedance or resonator template. They were obtained independently by direct binary metallization search and differ substantially in internal metal organization. Their electrical responses also exhibit distinct tradeoffs. Table 5 reports a common set of response metrics extracted from the uniform 51-point display sweep. The transition width is defined as

(13) $$\Delta f_{\mathrm{tr}}=f\;\left(\left|S_{21}\right|=0.2\right)-f\;\left(\left|S_{21}\right|=0.8\right),$$

evaluated along the main descending transition.

Pixelated LPF 1 provides the narrowest transition and strongest stopband attenuation among the three pixelated examples, whereas Pixelated LPF 2 provides the strongest worst-case passband transmission. Pixelated LPF 3 provides an intermediate passband level while maintaining stopband attenuation stronger than the classical reference. These results illustrate that distinct topology-free metallization patterns can emphasize different response characteristics under the same design domain and spectral constraints.

### 3.3. Comparison with the Classical Stepped-Impedance Reference

**Figure 9:**
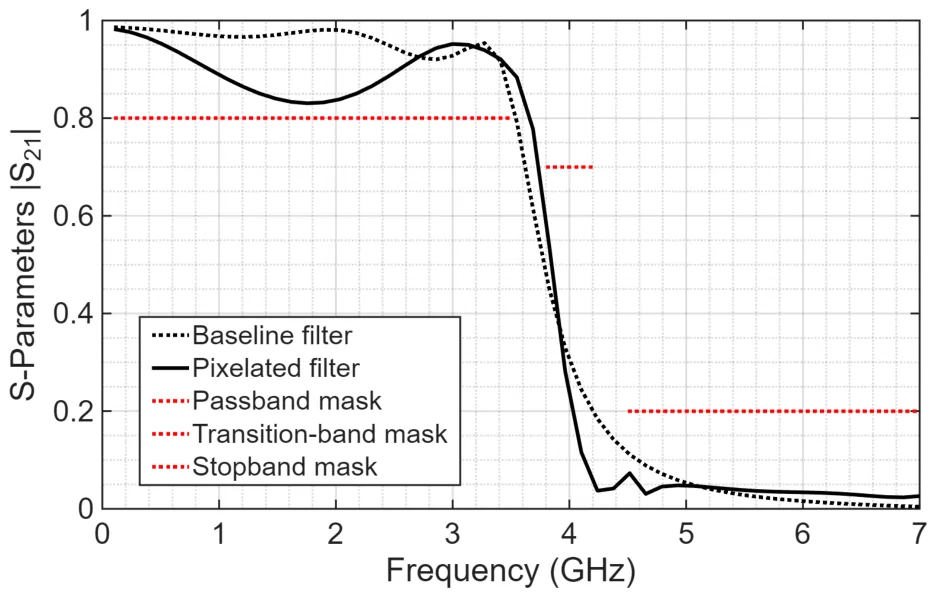
Comparison between the classical stepped-impedance reference (black dotted) and Pixelated LPF 1 (black solid) under comparable footprint conditions. Red dotted segments denote, over their respective frequency intervals, the passband, transition-band, and stopband masks. The comparison is intended to establish response-level competitiveness within a similar physical scale rather than universal superiority of the pixelated formulation.

The representative pixelated responses are competitive with the classical stepped-impedance reference under comparable footprint conditions, although the comparison does not indicate uniform superiority of either design paradigm. The classical filter retains the strongest worst-case passband transmission and return-loss performance in Table 5. Conversely, Pixelated LPF 1 exhibits both a narrower transition and stronger worst-case stopband attenuation than the reference. Pixelated LPF 3 also maintains stronger stopband attenuation than the reference while preserving a similar general cutoff region. Figure 9 directly compares the classical reference and Pixelated LPF 1 over the common display sweep.

The significance of this comparison is therefore topological rather than merely numerical. The pixelated solutions achieve valid LPF behavior without prescribing the internal arrangement of high- and low-impedance sections in advance. At the same time, the present benchmark remains deliberately limited: it compares the proposed formulation with one physically comparable classical reference and does not establish superiority over the broader state-of-the-art. A wider literature-based comparison and the implications for other microwave components are discussed separately in Section 4.

### 3.4. Structural Diversity of the Feasible Designs

**Figure 10:**
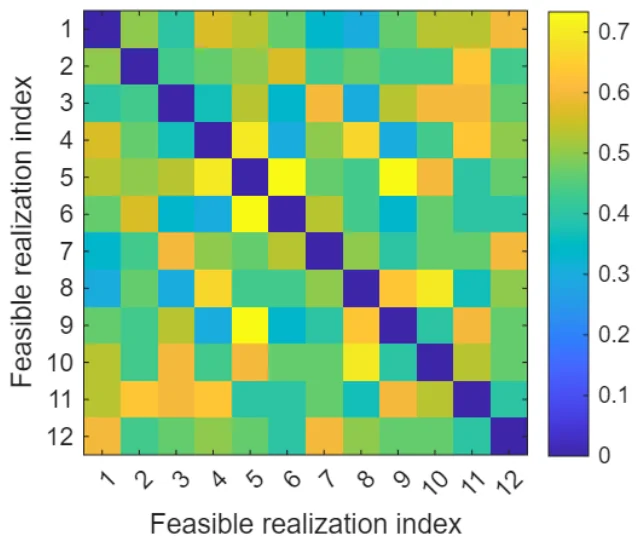
Normalized pairwise Hamming distance matrix for the 12 geometrically unique specification-compliant pixelated LPF realizations found under the present GA settings.

The 12 specification-compliant layouts found by the present GA runs were compared using pairwise Hamming distance. For two 30-bit designs, the Hamming distance is the number of pixel states that differ between their binary vectors. Figure 10 shows the resulting normalized pairwise distance matrix.

The pairwise distances range from 9 to 22 pixels, with a mean of 14.68 pixels, corresponding to a normalized mean of 0.489. Thus, two solutions in the recovered set differ, on average, in nearly half of the 30 binary states. This supports a high degree of geometric diversity among the designs found. However, a normalized Hamming distance of 0.489 is also close to the expected 0.5 value under a simple independent equiprobable binary model. The Hamming analysis therefore does not establish clustering, non-random organization, or a statistically significant departure from a random binary baseline.

This interpretation is further limited by the sampling process. The 12 layouts were generated by the same GA framework and each successful run terminated after the first solution satisfying Equation (11) was found. Consequently, the set should be viewed as a collection of diverse feasible outcomes found under the present GA settings rather than as an unbiased sample of the complete feasible manifold.

### 3.5. Pixel Activation Recurrence Across the Twelve Feasible Designs

**Figure 11:**
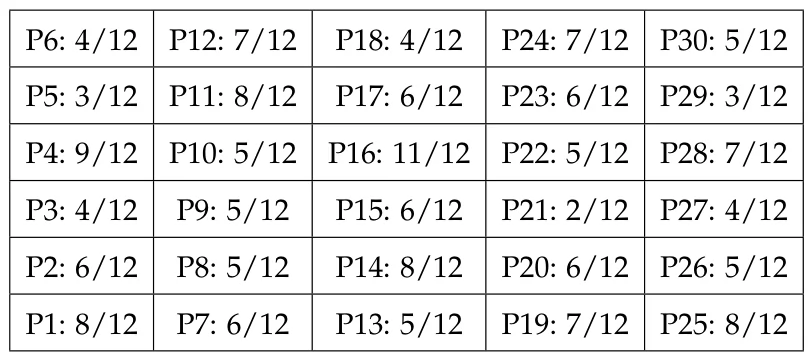
Spatial pixel-activation recurrence map across the 12 specification-compliant designs. Each entry gives the number of recovered layouts in which the corresponding pixel is active. Rows are displayed from physical row 6 at the top to row 1 at the bottom, matching the indexing orientation in Figure 2b.

Pixel activation frequency provides a complementary description of the 12 recovered solutions. Figure 11 reports, for each bit position, the number of layouts in which the pixel is active. The most recurrent pixel, P16, is present in 11 of the 12 solutions, whereas other locations are used much less frequently. Several additional positions occur in 7–9 designs, indicating recurrent use of portions of the interior domain within this particular solution set.

Because only 12 designs are available and all were produced by the same stochastic search procedure, these frequencies are descriptive rather than inferential. In particular, high activation frequency should not be interpreted by itself as evidence that a pixel is electromagnetically critical. Functional importance is examined separately through direct one-pixel perturbations in the next subsection.

### 3.6. Aggregate Single-Pixel Perturbation Analysis

To assess local functional sensitivity without relying on a single representative layout, one-pixel perturbation analysis was repeated for all 12 specification-compliant designs. For design $x_{j}$, pixel *i* is toggled while all other states remain unchanged:

(14) $$x_{j}^{\left(i\right)}=\left[x_{j,1},\ldots ,x_{j,i-1},1-x_{j,i},x_{j,i+1},\ldots ,x_{j,30}\right].$$

The corresponding sensitivity is

(15) $$\Delta F_{j,i}=F\;\left(x_{j}^{\left(i\right)}\right)-F\left(x_{j}\right).$$

All 12 baseline designs were independently re-evaluated and satisfy $F(x_{j})=0$; therefore, $\Delta F_{j,i}=F\left(x_{j}^{\left(i\right)}\right)$ for the present data set. Toggling all 30 pixels in all 12 layouts required 360 additional candidate evaluations, each using the original three band-specific full-wave sweeps.

Of the 360 perturbations, 59 (16.4%) remained strictly feasible with F=0, whereas 301 (83.6%) lost strict feasibility. A total of 116 perturbations produced F>1, and 53 produced F>10. The latter threshold is used here only as a descriptive marker of severe response collapse. Notably, all 53 severe cases correspond to removal of an active pixel (1→0); none result from metal addition (0→1). Across all evaluated flips, the 185 additions produced a mean ΔF of approximately 0.92 and a median of 0.28, whereas the 175 removals produced a mean of approximately 5.13 and a median of 0.57. Thus, removing an existing conductive element is substantially more damaging on average than adding a metal pixel.

**Figure 12:**
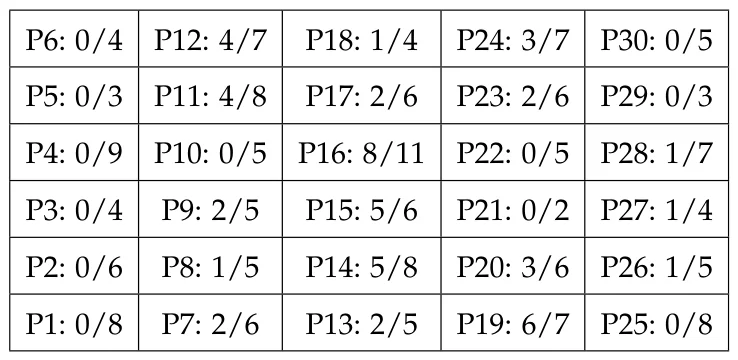
Spatial severe-collapse sensitivity map for active-pixel removal across the 12 feasible designs. Each entry reports the number of removals producing ΔF>10 divided by the number of recovered layouts in which that pixel was active. Rows follow the same physical orientation as Figure 2b; the result identifies topology-dependent sensitive regions without implying a universal conductive structure.

Figure 12 reports the conditional rate of severe collapse for active-pixel removal. The strongest rates are not confined to a single bit position: for example, P19 produces severe collapse in 6 of 7 active cases, P15 in 5 of 6, P16 in 8 of 11, and P14 in 5 of 8. At the same time, several frequently activated pixels exhibit no severe collapses when removed. Activation recurrence and perturbation sensitivity therefore provide distinct information.

The aggregate perturbation data support a more cautious structural interpretation than a single-layout sensitivity map. Functional dependence is strongly topology-dependent, yet several interior positions repeatedly exhibit high conditional sensitivity. It is therefore more appropriate to describe the recovered layouts as exhibiting topology-dependent sensitive conductive paths and regions, rather than to infer a single universal structural motif shared by all feasible designs.

### 3.7. Finite-Connectivity Regularization of Corner Contacts

The verified representative layouts contain diagonally adjacent metallized pixels that meet only at an ideal geometric point. Such point contacts are potentially fragile because their electromagnetic behavior can depend on geometry union and local discretization, while practical fabrication may either open the connection or create a bridge of finite width. The three representative layouts were therefore examined explicitly for pure diagonal contacts. Pixelated LPF 1 contains seven such contacts, three of which are graph-critical under a side-connected binary adjacency model; Pixelated LPF 2 contains three, all graph-critical; and Pixelated LPF 3 contains two, both graph-critical. The graph criterion is used only as a geometric diagnostic and does not replace full-wave EM evaluation.

All detected point contacts were regularized by finite conductive bridges rather than modification of only the graph-critical subset. Bridge widths were normalized to the feed-line width as $w_{b}/w_{\mathrm{line}}$ and initially evaluated at 0.05, 0.10, and 0.15. The results show that finite regularization is possible, but a single bridge width is not universally appropriate. In particular, refined-mesh evaluation of Pixelated LPF 1 shows that $w_{b}/w_{\mathrm{line}}=0.10$ preserves strict compliance, whereas 0.125 produces a small transition-band violation and further width increases progressively raise $|S_{21}|$ near 3.8 GHz. Conversely, Pixelated LPFs 2 and 3 require the wider tested value $w_{b}/w_{\mathrm{line}}=0.15$ to preserve strict compliance under the refined checks.

Table 6 summarizes the selected finite-bridge realizations among the tested configurations. In all three cases, both the dense 50 MHz sweep and the original objective-function frequency grid return zero penalty.

These results demonstrate that the ideal point-contact geometries can be converted into finite-connectivity realizations without necessarily destroying the intended LPF response. They also show that the regularization is topology-dependent: increasing bridge width can improve one layout while shifting a critical transition feature in another. The selected values should therefore be interpreted as feasible choices among the tested configurations rather than as globally optimal or universally transferable bridge dimensions.

### 3.8. Mesh-Refinement Assessment

Because narrow conductive connections can amplify discretization sensitivity, the bridge-regularized structures were re-evaluated under progressively refined meshes. The analysis is intentionally framed as a mesh-refinement assessment rather than a claim of strict mesh independence. For Pixelated LPF 1 at $w_{b}/w_{\mathrm{line}}=0.10$, the maximum pointwise change in $|S_{21}|$ decreases from 0.1279 for the base-to-finer comparison, to 0.1110 for finer-to-extra-fine, and to 0.0837 for extra-fine-to-the-finest-tested mesh. The corresponding RMS differences decrease from 0.0321 to 0.0281 and 0.0187. For the selected $w_{b}/w_{\mathrm{line}}=0.15$ configurations of Pixelated LPFs 2 and 3, the base-to-extra-fine differences are substantially smaller, with maximum deviations of 0.0414 and 0.0340 and RMS deviations of 0.0146 and 0.0116, respectively. The corresponding mesh-refinement differences are summarized in Table 7.

The decreasing differences for Pixelated LPF 1 indicate progressive stabilization with refinement, although the remaining variation is large enough that the structure should not be described as strictly mesh-independent. Pixelated LPFs 2 and 3 show smaller response changes over the tested refinement interval. Most importantly for the present manufacturability check, the final refined versions listed in Table 6 satisfy both the dense sweep and the exact optimization constraints.

### 3.9. Surface-Current Interpretation

**Figure 13:**
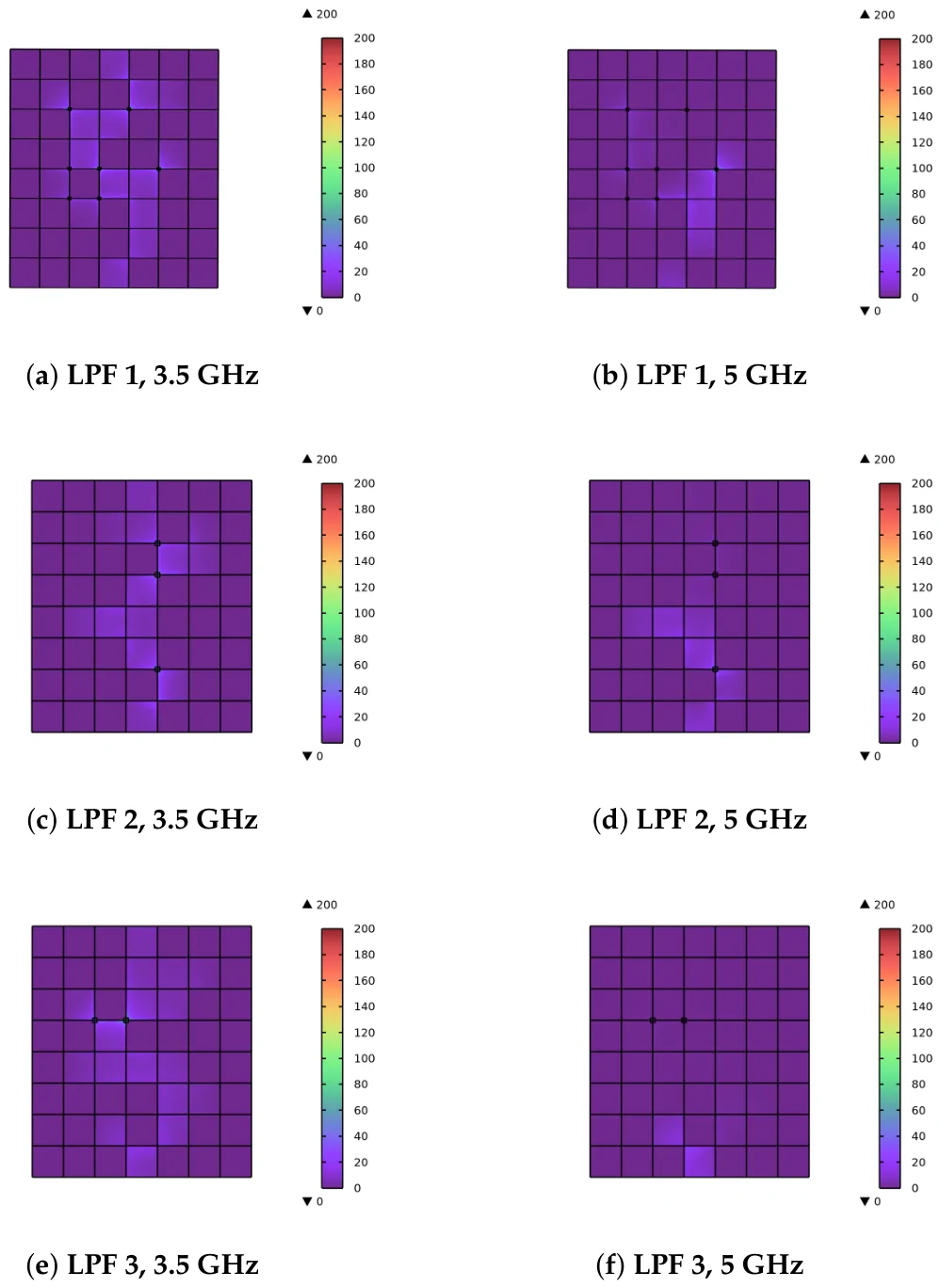
Surface-current-density norm for the final finite-bridge representatives at 3.5 GHz and 5 GHz. All panels use the same 0–200 A/m scale. At 3.5 GHz, the current is distributed through the conductive regions and finite-width connections that participate in the passband path. At 5 GHz, the current becomes substantially weaker and/or more localized, consistent with stopband suppression.

Surface-current-density maps were evaluated at 3.5 GHz and 5 GHz for the final bridge-regularized representatives. The same 0–200 A/m color scale is used for all six maps in Figure 13, enabling direct qualitative comparison between the passband-edge and stopband cases.

At 3.5 GHz, the three layouts exhibit topology-specific but clearly connected current distributions in which the finite bridges participate in the conductive path. Pixelated LPF 2 shows a particularly pronounced central chain of current-carrying connections, whereas Pixelated LPF 3 concentrates the current around its two upper finite connections. At 5 GHz, the response becomes markedly weaker and more localized over large portions of each pixelated region. These maps do not imply that the current flows exclusively through the bridges; rather, they confirm that the regularized connections are electromagnetically active elements of the passband structures and that the current distribution changes consistently with the transition to stopband behavior.

#### Relation Between Structural Recurrence, Perturbation Sensitivity, and Driven Current

The activation, perturbation, and current-density results describe different aspects of the recovered structures and should therefore be interpreted jointly rather than collapsed into a single pixel-importance measure. Across the 12-design ensemble, P16 is both the most recurrent pixel (11/12 layouts) and one of the most sensitive active locations, with severe collapse after removal in 8/11 active cases. By contrast, P4 is also highly recurrent (9/12) but produces no severe-collapse event in its nine active cases, whereas P19 is less recurrent (7/12) but produces severe collapse in 6/7 active cases. Thus, recurrence alone is not a monotonic proxy for functional criticality.

Figure 13 adds frequency-dependent electromagnetic context to this structural picture. At 3.5 GHz, the current is carried along topology-specific connected metallization paths and through the finite-width connections, whereas at 5 GHz, the current distribution becomes weaker and more spatially localized. The current-carrying regions are therefore not expected to coincide with a frequency-independent set of “important” pixels. This observation is consistent with the aggregate perturbation result: the role of a metallized location depends on the complete surrounding topology and on the operating frequency. In this sense, the recurrence map identifies where metal repeatedly appears in the solutions found, the one-pixel experiment identifies where local state changes can strongly disrupt compliance, and the driven-current maps show how representative complete layouts support or suppress transmission at two distinct frequencies.

A pixel-wise statistical correlation coefficient between current density and activation recurrence is intentionally not reported. The recurrence and perturbation statistics span all 12 nominal feasible layouts, whereas the available current maps correspond to the three final bridge-regularized representatives. Combining these unequal ensembles into a numerical correlation would imply a level of comparability not supported by the present data. The supported conclusion is therefore qualitative but direct: recurrent activation, local perturbation sensitivity, and driven current participation are related descriptors, yet none of them alone defines a universal electromagnetic backbone for the topology-free solutions.

No eigenmode decomposition or local impedance extraction is introduced in the present study. Such analyses could provide a deeper reduced-order interpretation of individual layouts, including representation-dependent inductive or capacitive roles, but they would require an additional model-identification and validation stage beyond the response-based full-wave synthesis investigated here. Introducing an unvalidated equivalent-network decomposition solely to assign canonical circuit roles to free-form distributed metallization could over-interpret the present results. Modal and impedance-based physical decomposition are therefore identified as valuable future extensions rather than prerequisites for the numerical claims made in this work.

## 4. Discussion

### 4.1. Implications of Topology-Free Design Under Comparable Footprint

The results of this study suggest that topology-free pixelated EM design can serve as a meaningful alternative framework for planar microstrip LPF synthesis when the available physical footprint is constrained but not aggressively minimized. In the present formulation, the main point is not that pixelation guarantees universally superior responses, nor that classical stepped-impedance synthesis should be displaced. Rather, the results indicate that once topology-specific assumptions are relaxed, the same general physical scale can support more than one functional low-pass realization, including layouts whose responses remain competitive with a conventional reference and, in some representative cases, exhibit sharper transitions or stronger stopband suppression. This observation is relevant because it reframes the role of pixelated EM design. In much of the recent literature, pixelated structures are often motivated by miniaturization or by the search for highly unconventional layouts.

The present work suggests an additional perspective: even when extreme area reduction is not the primary objective, topological freedom itself can be a useful design resource. Under a comparable footprint, the binary metallization domain can host responses that are not trivially inherited from a predefined stepped-impedance architecture yet still satisfy the same electrical requirements. In that sense, the approach expands the space of admissible microstrip filter realizations without requiring that the designer commits in advance to a particular circuit topology.

For quantitative context, Table 8 broadens the comparison beyond the three LPF examples considered previously and includes recent free-form or inverse-designed microwave filters that are directly comparable in function or closely related in methodology. The table deliberately separates filter class, representation, physical scale, passband performance, rejection/selectivity, and source-reported normalized metrics. This avoids collapsing dissimilar objectives into a single ranking. In particular, the studies span fixed low-pass, tunable low-pass, wideband, dual-band, bandpass, and notch responses; they also use different substrates, cutoff or center frequencies, stopband definitions, normalization wavelengths, and design objectives. Consequently, a single composite figure of merit combining insertion loss, roll-off, and size would require arbitrary cross-study weights and, in several cases, quantities that are not reported under compatible definitions. We therefore report normalized size or source-defined FOM values when they are available, rather than constructing a new universal score from nonuniform data.

Among the most directly comparable LPF studies, Zhang and Wang [40] reported a measured two-bit fragment-type LPF with a normalized circuit area of $0.019\lambda_{g}^{2}$, insertion loss not exceeding 0.3 dB, a roll-off rate of 123.3 dB/GHz, a 20 dB relative stopband bandwidth of 1.65, and a source-defined FOM of 21060. Zou et al. [10] reported a measured pixelated LPF occupying $0.0935\lambda_{g}\times 0.0935\lambda_{g}$ (normalized area $\approx 0.00874\lambda_{g}^{2}$), with approximately 0.3 dB insertion loss and more than 20 dB out-of-band suppression. Zhang, Xu, and Jiang [38] demonstrated inverse-designed tunable pixelated LPFs with customized tuning ranges between 5 and 10 GHz and a measured rejection of approximately 17 dB over a wide stopband extending to 28 GHz. More recently, Zhou et al. [41] reported a measured 13×13 pixelated LPF with a physical scale of approximately 0.3λ×0.3λ at the 3 dB frequency, insertion loss below 0.36 dB up to 7 GHz, and more than 20 dB suppression beyond 9.5 GHz.

The broader inverse-design literature also provides useful methodological context even when the filter response is not a fixed LPF. Gomez et al. [37] used a 48×32 pixelated region of 9.6×6.4 mm for inverse-designed notch/bandpass filters and reported measured structures with approximately 35 dB notch-depth improvement and about 10% bandwidth enhancement after introducing shorted-stub features. Wang et al. [39] combined a generative model with a GA for inverse-designed single- and dual-band microstrip filters; measured prototypes showed insertion loss below 1.23 dB for the single-band example and below 1.19 dB for the dual-band example, with return loss exceeding 15.3 dB and 10.8 dB, respectively. These studies emphasize miniaturization, tunability, rapid inverse synthesis, or specific spectral functions. In contrast, the present work deliberately fixes a comparable physical envelope relative to a classical stepped-impedance reference and investigates whether topological freedom alone can yield multiple specification-compliant LPF realizations, together with aggregate sensitivity and finite-connectivity verification.

The expanded comparison makes two points explicit. First, the present numerical results are not intended to outperform every recent measured filter on insertion loss, normalized area, or roll-off. Several published inverse-designed LPFs achieve lower insertion loss or stronger normalized miniaturization than the present examples. Second, those studies optimize different objectives and often introduce additional design resources such as multi-bit fragments, varactors, neural inverse models, shorted stubs, or higher-resolution pixel domains. The distinctive question examined here is therefore not a universal performance ranking, but whether a topology-free binary metallization domain can produce multiple useful low-pass realizations while the available physical footprint is held comparable to a classical reference. This distinction is also why a source-independent scalar FOM is not used: any such score would mix unlike electrical specifications and geometric normalizations and could obscure, rather than clarify, the methodological comparison.

The structural analyses of Section 3.4, Section 3.5, Section 3.6, Section 3.7, Section 3.8 and Section 3.9 further suggest that this freedom is not equivalent to complete arbitrariness. The recovered solutions exhibit high geometric diversity together with nonuniform activation recurrence. Multiple geometrically distinct layouts are possible, but the Hamming-distance statistics do not establish clustering or a departure from a simple random binary baseline. Some regions are activated more frequently, while the aggregate one-pixel perturbation analysis shows that functional sensitivity is strongly topology-dependent and that activation recurrence does not by itself imply criticality. The finite-bridge and surface-current analyses further show that local connections can be electromagnetically active in the passband response, although their suitable regularization depends on the particular topology. Such a picture is qualitatively different from the logic of classical synthesis, where functionality is usually tied to an explicitly prescribed arrangement of sections or resonators; however, the present data do not support a universal structural template for the complete feasible manifold.

From a broader design perspective, these results support a balanced interpretation. Classical filter synthesis remains highly valuable because of its physical transparency, mature procedures, and direct control over well-understood topologies. However, the present study shows that topology-free pixelated EM design can uncover additional realizations that may not be obvious from conventional intuition alone. This makes the framework potentially useful not only for obtaining alternative layouts under constrained footprint conditions, but also for studying how distributed low-pass behavior can emerge from non-prescribed metallization patterns.

### 4.2. Limitations and Future Work

The present study also has important limitations that should be stated explicitly. First, the investigation is restricted to a single class of device, namely a microstrip low-pass filter, and to one specific comparable-footprint design domain. Although this is sufficient to demonstrate the basic feasibility of the topology-free approach in a controlled setting, it does not by itself establish generality across other filter classes, bandwidth regimes, or substrate technologies. Extension to bandpass, dual-band, wideband, multilayer, resonator-based, or strongly coupled structures would require additional study. The binary metallization concept itself is not tied to the low-pass response: related pixelated and inverse-design studies have already been demonstrated for waveguide filters, couplers, frequency-selective structures, and resonant devices [20,21,28,31,42]. The present formulation could, therefore, be transferred in principle by redefining the EM domain, fixed ports or excitations, and response-based objective for the target component, although such transfer is not demonstrated here.

Second, the manuscript is intentionally scoped as a simulation-based EM modeling and analysis study. Experimental fabrication and measurement are therefore not part of the evidence used to support the present claims, which are restricted to modeled EM behavior, numerical feasibility, and structural interpretation of the recovered layouts. Geometric manufacturability was examined numerically through finite-width regularization of ideal corner contacts, bridge-width sweeps, and refined-mesh reevaluation; these analyses test whether idealized connectivity can be represented by finite conductive features within the EM model, but they are not presented as a substitute for experimental validation. Conductor loss, dimensional tolerances, substrate variation, connector transitions, and fabrication-induced perturbations were not explored here in a systematic experimental setting, and the PEC model does not capture finite conductor thickness or ohmic loss. These effects belong to a subsequent hardware-validation stage and may become important when pixel sizes are small or when narrow conductive necks appear in feasible layouts.

Third, the search process is budget-constrained and stochastic. The feasible layouts reported here should therefore be viewed as representative outcomes of the adopted exploration strategy rather than as exhaustive characterization of the entire feasible set. The structural analysis reported here is based on 12 specification-compliant realizations found under the adopted budget-constrained stochastic search. Similarly, the Hamming-distance analysis demonstrates structural diversity within the set of realizations found, but it does not constitute a complete map of the feasible manifold. The aggregate one-pixel perturbation analysis spans all 12 recovered feasible designs, but it remains a local one-bit analysis conditioned on this finite set and should not be overextended into a universal statement about all feasible layouts in the domain. Scalability is also a practical limitation: for *N* binary design variables, the nominal design space contains $2^{N}$ patterns, while each direct objective evaluation still requires the three band-specific full-wave EM sweeps defined in Section 2.3. Consequently, simply increasing the pixel resolution while retaining the present direct-GA/full-wave strategy is not expected to remain computationally practical at substantially larger *N*.

A concrete extension would be a hierarchical multiresolution search. The optimization could begin on a coarse binary grid with substantially fewer design variables. After a feasible or near-feasible coarse layout is identified, only selected regions would be subdivided into finer pixels, while the remaining regions retain their coarse representation. Candidate regions for refinement could be selected from metallization interfaces, large local one-pixel sensitivities, or persistent current-carrying regions. The refined problem would then inherit the coarse solution as its initial structural seed, and the process could be repeated until the desired spatial resolution is reached. Such a coarse-to-fine procedure would preserve the topology-free character of the formulation while replacing one uniformly high-dimensional binary search with a sequence of smaller, adaptively refined searches. The recurrence and one-pixel sensitivity information reported in Section 3.5 and Section 3.6 provides quantities that could be used as refinement indicators in such a future implementation.

A complementary route is to reduce the number of fine full-wave evaluations through surrogate-assisted optimization. In a space-mapping formulation, a computationally cheaper coarse EM model can be calibrated against the fine full-wave model and used for most optimization steps, reserving fine-model evaluations for correction and validation [11,12]. Alternatively, forward neural surrogates or reduced-dimensional inverse models can learn the relationship between geometry and microwave response, and screen large numbers of candidate layouts before selected candidates are verified by the full-wave solver [15,18]. Recent surrogate-assisted and data-driven topology-design studies further illustrate this possibility for pixelated EM structures [29,33,34]. In an adaptive implementation, newly computed full-wave samples could be returned to the surrogate during the search, concentrating expensive simulations on promising or poorly predicted regions of the design space. These strategies are proposed here as specific scalability directions; their computational gains and convergence behavior are not evaluated in the present 30-variable study.

Fourth, the physical interpretation in the present work remains based on driven full-wave surface-current maps and direct perturbation of the recovered binary layouts. It does not construct an eigenmode description or an extracted equivalent impedance network. Those tools could deepen the physical interpretation of a particular optimized geometry, but they would introduce an additional reduced-order modelling problem whose representation and validation are distinct from the topology-free synthesis question addressed here. Their systematic use across multiple optimized layouts is therefore left for future work.

These limitations point naturally to several directions for future work. The first extension would be to incorporate experimental validation of representative topology-free pixelated LPFs. A second would be to examine robustness under fabrication uncertainty, conductor loss, substrate variation, and dimensional tolerances beyond the finite-bridge regularization considered here. A third would be to repeat the structural analysis across other filter classes, resonators, and larger design domains to assess how diversity, recurrence, perturbation sensitivity, and search cost evolve with device complexity. Finally, the hierarchical and surrogate-assisted scalability pathways outlined above should be evaluated on larger pixel domains to quantify the reduction in fine full-wave evaluations, the preservation of feasible solutions across refinement levels, and the resulting convergence behavior without reimposing a conventional filter template.

## 5. Conclusions

This paper has investigated a topology-free pixelated EM modeling and analysis framework for microstrip low-pass filters under a physical footprint comparable to that of a classical stepped-impedance reference. At the full-wave modeling level, the study shows that specification-compliant pixelated LPF layouts can be obtained without prescribing an internal filter topology in advance, and that representative layouts remain competitive with the classical reference under the same general physical scale. Some pixelated layouts exhibit sharper transitions and/or stronger stopband attenuation in simulation, while the classical stepped-impedance filter retains the strongest worst-case passband transmission.

The results also show that the topology-free pixelated domain does not yield only a single obvious geometry. Across 20 independent runs, 12 geometrically distinct specification-compliant layouts were identified, and the pairwise Hamming analysis confirmed a high degree of geometric diversity within the recovered set. Pixel activation recurrence and the aggregate one-pixel perturbation analysis further show that activation frequency and functional sensitivity provide distinct, topology-dependent information rather than evidence of a universal pixel-level structural motif. In addition, ideal corner contacts in the representative layouts could be regularized using finite-width conductive bridges while preserving strict compliance after refined-mesh verification, although suitable bridge width depends on the individual topology.

Taken together, these findings support a measured conclusion: topology-free pixelated EM modeling and analysis is not merely a route to unusual layouts, nor only a tool for aggressive miniaturization. Under comparable footprint constraints, it provides a simulation-based framework for exploring non-obvious microstrip LPF layouts that remain electromagnetically meaningful within the adopted full-wave model, structurally interpretable, and amenable to finite-connectivity regularization. The present conclusions are therefore restricted to numerical EM feasibility and structural analysis; experimental hardware validation remains a distinct subsequent stage.

## Figures and Tables

**Table 1:** Dimensions of the reference stepped-impedance LPF.

Section	Function	$Z_{0}$ (Ω)	Width (mm)	Length (mm)
Feed IN	50 Ω feed line	50	3.048	0.800
$L_{1}$	High-impedance line	≈90	1.11	5.184
$C_{2}$	Low-impedance line	≈20	12.00	2.880
$L_{3}$	High-impedance line	≈90	1.11	6.560
$C_{4}$	Low-impedance line	≈20	12.00	3.008
$L_{5}$	High-impedance line	≈90	1.11	6.392
$C_{6}$	Low-impedance line	≈20	12.00	2.080
Feed OUT	50 Ω feed line	50	3.048	0.800

**Table 2:** Geometric and encoding parameters of the pixelated design domain.

Category	Parameter	Value
Geometry	Total discretized grid	8×7 pixels
Geometry	Active design region	6×5 pixels
Geometry	Pixel side length	$W_{0}=3.048$ mm
Geometry	Envelope length	$8W_{0}=24.384$ mm
Geometry	Envelope width	$7W_{0}=21.336$ mm
Constraints	Fixed port pixels	2 (non-optimizable)
Constraints	Boundary region	Non-metallizable
Encoding	Number of design variables	30
Encoding	Design variable type	Binary ($x_{i}\in \left\{0,1\right\}$)

**Table 3:** Genetic algorithm configuration used in optimization experiments.

Category	Parameter	Value
Encoding	Representation	Bitstring, $x\in {\left\{0,1\right\}}^{30}$
Population	Size	15
Population	Elite count	1
Selection	Operator	Stochastic uniform
Crossover	Operator	Scattered
Crossover	Fraction	0.75
Mutation	Operator	Uniform bit-flip
Mutation	Probability $p_{m}$	0.05, 0.10
Stopping	Function tolerance	$10^{-5}$
Stopping	Maximum stall generations	15
Budget	Candidate EM evaluations $N_{\mathrm{EM}}$	300
Experiment	Independent runs	10 per mutation setting

**Table 4:** Search outcomes under the two mutation settings. Confidence intervals are 95% Wilson intervals for the empirical success probability. First F=0 statistics are reported only over successful runs.

Mutation Probability	Successful Runs	Success Rate	95% CI	First F=0 Eval., Median [Range]
$p_{m}=0.05$	6/10	60%	31.3–83.2%	270 [180–300]
$p_{m}=0.10$	6/10	60%	31.3–83.2%	135 [45–195]
All runs	12/20	60%	38.7–78.1%	187.5 [45–300]

**Table 5:** Electrical comparison of the classical stepped-impedance reference and the three verified topology-free pixelated LPF realizations. Metrics are extracted from the common uniform 51-point sweep over 0.1–7 GHz.

Design	Min $|S_{21}|$ PB	IL_max_ (dB)	Max $|S_{11}|$ PB	RL_min_ (dB)	Max $|S_{21}|$ SB	$A_{\mathrm{SB},\min}$ (dB)	*f*_3dB_ (GHz)	$\Delta f_{\mathrm{tr}}$ (GHz)
Reference filter	0.9159	0.763	0.3466	9.205	0.1114	19.066	3.616	0.663
Pixelated LPF 1	0.8304	1.614	0.5364	5.410	0.0729	22.748	3.729	0.373
Pixelated LPF 2	0.9006	0.910	0.4083	7.781	0.1821	14.794	3.756	0.591
Pixelated LPF 3	0.8861	1.051	0.4388	7.155	0.0960	20.359	3.689	0.600

**Table 6:** Selected finite-bridge configurations among the tested widths after refined-mesh verification. The reported band extrema are extracted from the dense 50 MHz sweep over 0.1–7 GHz.

Design	Diagonal Contacts	Graph-Critical	$w_{b}/w_{\mathrm{Line}}$	Final Mesh Check	Min PB	Max TR	Max SB	$F_{\mathrm{dense}}$	$F_{\mathrm{exact}}$
Pixelated LPF 1	7	3	0.10	Finest tested	0.8928	0.6863	0.0606	0	0
Pixelated LPF 2	3	3	0.15	Extra-fine	0.8026	0.5422	0.1529	0	0
Pixelated LPF 3	2	2	0.15	Extra-fine	0.8077	0.4399	0.0828	0	0

**Table 7:** Mesh-refinement response differences for the selected bridge-regularized configurations, evaluated over the dense 0.1–7 GHz sweep with 50 MHz spacing.

Design	$w_{b}/w_{\mathrm{line}}$	Mesh Comparison	max $|\Delta S_{21}|$	RMS $\Delta S_{21}$
LPF 1	0.10	Base → Finer	0.1279	0.0321
LPF 1	0.10	Finer → Extra-fine	0.1110	0.0281
LPF 1	0.10	Extra-fine → Finest tested	0.0837	0.0187
LPF 2	0.15	Base → Extra-fine	0.0414	0.0146
LPF 3	0.15	Base → Extra-fine	0.0340	0.0116

**Table 8:** Expanded quantitative context for recent free-form or inverse-designed planar microwave filters. Normalized dimensions and FOM values are retained using the definitions reported by each source. “–” indicates that a directly comparable quantity was not reported or would require a nonuniform cross-study definition. The table is intended for positioning rather than direct performance ranking.

Study	Filter/Design Approach	Scale	Reported Electrical Performance	Normalized/Source-Specific Metric	Validation
Zhang and Wang [40]	LPF; two-bit FTS optimization	Source-normalized area	IL ≤0.3 dB; $f_{3\;\mathrm{dB}}=2.0$ GHz; roll-off 123.3 dB/GHz; 20 dB RSB = 1.65	$0.019\lambda_{g}^{2}$; reported FOM = 21,060	Measured
Zou et al. [10]	LPF; pixelated optimization	Source-normalized footprint	IL ≈0.3 dB; out-of-band suppression >20 dB	$0.0935\lambda_{g}\times 0.0935\lambda_{g}$; area $\approx 0.00874\lambda_{g}^{2}$	Measured
Zhang et al. [38]	Tunable LPF; pixelated GAN + transfer learning	Source-specific tunable layouts	Customized tuning ranges within 5–10 GHz; measured rejection ≈17 dB over a wide stopband to 28 GHz	–	Measured
Gomez et al. [37]	Notch/BPF; inverse-designed pixels + shorted stubs	48×32 pixels; 9.6×6.4 mm	∼35 dB notch-depth improvement and ∼10% bandwidth enhancement after stub integration	–	Measured
Wang et al. [39]	Single/dual-band; generative model + GA	26 × 26 mm / 42 × 42 mm	IL <1.23 / <1.19 dB; RL >15.3 / >10.8 dB; transmission zeros flanking passband(s)	Inverse-design MAE 0.039 dB; 3.6 min/1000 iter	Measured
Zhou et al. [41]	LPF; 13×13 pixels + DL/GA	∼0.3λ×0.3λ	IL <0.36 dB up to 7 GHz; suppression >20 dB beyond 9.5 GHz	Area $∼0.09\lambda^{2}$	Measured
This work	LPF; 6×5 binary pixels + direct full-wave GA	24.384 × 21.336 mm; comparable to reference	IL_max_ = 0.910–1.614 dB; $A_{\mathrm{SB},\min}=14.794$–22.748 dB; $\Delta f_{\mathrm{tr}}=0.373$–0.600 GHz	Fixed-envelope benchmark; no universal FOM imposed	Full-wave EM

## Data Availability

The raw data supporting the conclusions of this article will be made available by the authors upon request.
